# Three-Dimensional Performance Evaluation of Hemispherical Coriolis Vibratory Gyroscopes

**DOI:** 10.3390/mi14020254

**Published:** 2023-01-19

**Authors:** Mehrdad Mahmoudian, Joel Filho, Rui Melicio, Eduardo Rodrigues, Mojgan Ghanbari, Paulo Gordo

**Affiliations:** 1 Department of Engineering and Technology, Apadana Institute of Higher Education, Shiraz 71789, Iran; 2 Instituto de Astrofísica e Ciências do Espaço, Departamento de Fisíca, Universidade de Coimbra, 3040-004 Coimbra, Portugal; 3 CENTRA, Faculdade de Ciências, Universidade de Lisboa, 1749-016 Lisboa, Portugal; 4 IDMEC, Instituto Superior Técnico, Universidade de Lisboa, 1049-001 Lisboa, Portugal; 5 ICT, Universidade de Évora, 7002-554 Évora, Portugal; 6 INESC-ID, Sustainable Power Systems Group, Instituto Superior Técnico, Universidade de Lisboa, 1049-001 Lisboa, Portugal; 7 Department of Electronics, Telecommunications and Informatics (DETI) University of Aveiro, 3810-193 Aveiro, Portugal

**Keywords:** Coriolis force, performance evaluation, hemispherical vibratory gyro, aeronautics applications, space applications, payload, spacecraft

## Abstract

In this paper, the oscillation patterns and characteristics of gyroscopic reaction to rotation-induced Coriolis force and phase relations are reviewed by examining the main principles of operation of Coriolis vibratory gyroscopes based on the dynamic relations and proposed improvements in performance using parameter changes. Coriolis vibratory gyroscopes (CVGs) are among the most modern applicable gyroscopes in position detection that have replaced traditional gyroscopes due to some great features of the design of vibrating proof mass and elastic suspension. Given the key characteristics of capacitive versus piezoelectric excitation technologies for determining the vibration type in sensors, their operating principles and equations have completely changed. Therefore, two-dimensional finite element analysis is required to evaluate their optimal performance. Since the sensor space is constantly vibrating, a general equation is presented in this paper to explain the impact of parameters on the frequency of different operating modes. The main purposes of building vibrating gyroscopes are replacing the constant spinning of the rotor with a vibrating structure and utilizing the Coriolis effect, based on which the secondary motion of the sensitive object is generated according to the external angular velocity.

## 1. Introduction

In recent years, gyroscopes have attracted the attention of many researchers, and vibratory gyroscopes are a fairly new scientific field. What has made these gyroscopes so attractive is not only their improved accuracy and their application in aeronautics and space technologies but also their extremely low cost, and small size and dimensions [1]. The low cost of these gyroscopes has made their application in so many new fields possible, including the automotive industry and automobile control, robotics, the control of cameras and binoculars, telescopes, gamepads and even toys [2]. A novel category of vibratory gyros with noteworthy applications named hemispherical resonator gyroscopes (HRGs) are much cheaper and take up very little space compared with mechanical and optical gyroscopes, which are bulky and expensive [3,4]. Early efforts to design this type of gyroscope began in the military industry in areas such as rocket stability and guidance, and smart ammunition, but recently, gyroscopes have also been utilized in non-military industries, such as the automotive industry (advanced braking system to prevent sliding) and handheld cameras and robotics [5,6,7]. In general, with advances in technology, building smaller, cheaper, more accurate and widely used vibratory gyroscopes has become more possible. The Coriolis effect only exists when a rotating frame of reference is used. The Coriolis effect acts like a real force in a rotating frame. However, the Coriolis force is an inertial force and is not proportional to the original position of the object. In some cases, the electromagnetic or atomic force is also considered a type of Coriolis force. From an analytical point of view, the Coriolis force is necessary when using Newton’s second law in a rotating frame, but this force does not act on an inertial frame of reference with zero acceleration [8].

A rotating frame (Earth) in the atmosphere is a natural frame (body), which explains the deflection of moving currents relative to a hypothetical, non-rotating, inertial frame without Coriolis force. Over long distances, the direction of artillery is affected by the Coriolis force. Examples are described in more detail below. Coriolis acceleration happens due to two types of changes in rotational velocity:

- The first one is a change in the velocity of the object over time. At different times, equal or different velocities might be observed in a rotating frame. In inertial frames, where the common laws of physics apply, the pseudo acceleration is proportional to the angular velocity of the reference frame (velocity changes direction in the coordinate axis) and the velocity of the object on a vertical plane around the axis of rotation. The minus sign is derived from the conventional definition of the cross product (right-hand rule) and the notation convention for angular velocity vectors.
- The second one is a change in space velocity. Different points have different velocities in a rotating coordinate axis (such as the inertial reference frame). In other words, a change in the position of an object needs to have accelerated linear motion, because the velocity changes by equal amounts from one point to another in the coordinate system. This effect (the Coriolis force) is proportional to the angular velocity (which determines the relative velocity of two different points in the rotating reference frame) and the velocity component on a vertical plane around the axis of rotation (which determines how fast it moves between points).

Some of the important advantages of these gyroscopes that have led researchers to implement them in rockets and aerospace industry are as follows [9,10]:

1. They have a short start-up time.
2. They do not need bearings due to the lack of a rotating shaft.
3. They do not need an engine.
4. When designed in an effective way, they show high durability and do not need maintenance or repair.
5. They are much smaller and lighter than traditional gyroscopes.
6. Low cost is one of the most important advantages of these gyroscopes.
7. They have a simpler manufacturing process.
8. They have low energy consumption.

The first vibratory gyroscopes were developed in the early 1980s based on the angular velocity [11]. Piezoelectric quartz, used in these types of gyroscopes, showed high efficiency and quality at atmospheric pressure [12]. In addition to this, the Japanese company Murata introduced two low-cost designs in the 1990s [13]. In one of the designs, a steel-beam pattern with a triangular cross-section is used, excited and sensed by piezoelectric elements connected to the beam surfaces. The second resonator is designed as a piezoelectric rectangle. In both designs, the resonators vibrate like free beams in the first mode of vibration, and the supports are placed at the nodal points. Gyroscopic designs based on string resonators, vibrating beams and pendulums are sensitive to linear acceleration. Defects can be eliminated using a symmetrical resonator, such as a diapason (known as tuning fork). The first design of the tuning fork was presented in [14]. In this design, the Coriolis force from the rotation of the vibrating prongs around the longitudinal axis of the tuning fork causes a basic torsional oscillation, and its amplitude is proportional to the applied angular velocity. The design is costly and bulky, and perhaps, this was the main reason for the inefficiency of the original designs of vibratory gyroscopes, which was eliminated by micromachining and reducing their dimensions. The main step in this field was taken by a German company [15].

**Figure 1:**
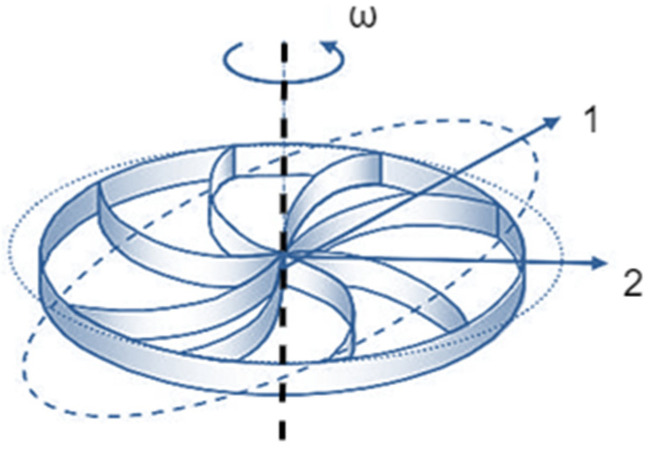
Circular CVG.

HRG technology exhibits highly competitive features compared with optical gyroscopes due to their low cost, small size and high efficiency. In [16], a Coriolis vibratory gyroscope with some developments in motion equations, where the deformation in the excitation frequency and damping axes are also taken into account, is presented. The gyroscopic vibrations studied in this reference, along with the changes in the vibrating object, have been verified based on the dynamic equations provided in the standard [17]. However, the calculation of the control factors of HRGs has not been clearly assessed, and the signal excitation and detection patterns also remain unclear. The authors present dynamic equations for a Coriolis vibratory gyro CVG (see Figure 1) in [1,18] and mathematically analyze its dynamic excitation and detection; however, as in reference [19], the calculation of its control factors and parameters is not reliable nor solid. In reference [20], dynamic equations are developed for a CVG based on Lagrange equations, but the vibrations in the resonator shell are performed in the absence of the excitation system and only using the characteristic equation. In [21], the hemispherical shell of a gyroscope is modeled with a particle, and a two-dimensional analysis of oscillation damping is also provided. The control scheme of signal detection is also presented, but the equivalent mass of the oscillating device is not calculated, and its three-dimensional analysis is overlooked. In reference [22], the CVG is equivalent to a loop, and the impact of important parameters such as the quality factor (Q) of the resonator in the model is examined (Figure 1); however, the dynamic effects of gyroscopes and their equations of state are overlooked. In reference [23], the equations of motion of HRGs are provided using the Galerkin method, but the analyses presented to evaluate the resonance frequency are inaccurate, and the theoretical results differ from the laboratory results in references [24,25] and the finite element outputs in [24,25,26,27]. Therefore, the presented equations are approximations that are only used in high-speed, surface analyses. In references [28,29], the equations of state for HRGs are provided based on Lagrange equations, but the control error refers to the output results in [30], and the distortion of parameters along the frequency and damping axes is still incomprehensible. Table 1 [1] presents a comparison among different HRG technologies and their functions.

In this paper, we examine the changes created in the motion and position of an HRG and present the equations. First, the natural frequencies in different modes are calculated; then, the gyroscope is designed. Next, the voltage is measured using the pickoff sensors around the frequencies of oscillation. Then, the displacement at the location of the pickoff electrodes, along with the changes in their size and material, is analyzed. In the discussion and conclusion section, a comparison is made among piezoelectric and capacitive gyroscopes, and combined piezocapacitive gyroscopes. Sensitivity analyses of the physical and geometric properties of the gyroscope and their impact on changes in resonance frequency and output voltage using pickoff sensors are also presented in the last section of the article.

## 2. HRG Formulation

All vibratory gyroscopes operate based on the Coriolis effect. Vibratory gyroscopes have non-rotating components that use Coriolis acceleration to measure the angular rotation rate of inertia. The Coriolis acceleration caused by the rotation of the reference frame is used to describe the rotational motion of the reference frame and to calculate the axial motion. The Coriolis effect exists in phenomena with complex rotation, including the flow of air above the Earth’s surface in the northern and southern hemispheres.

**Figure 2:**
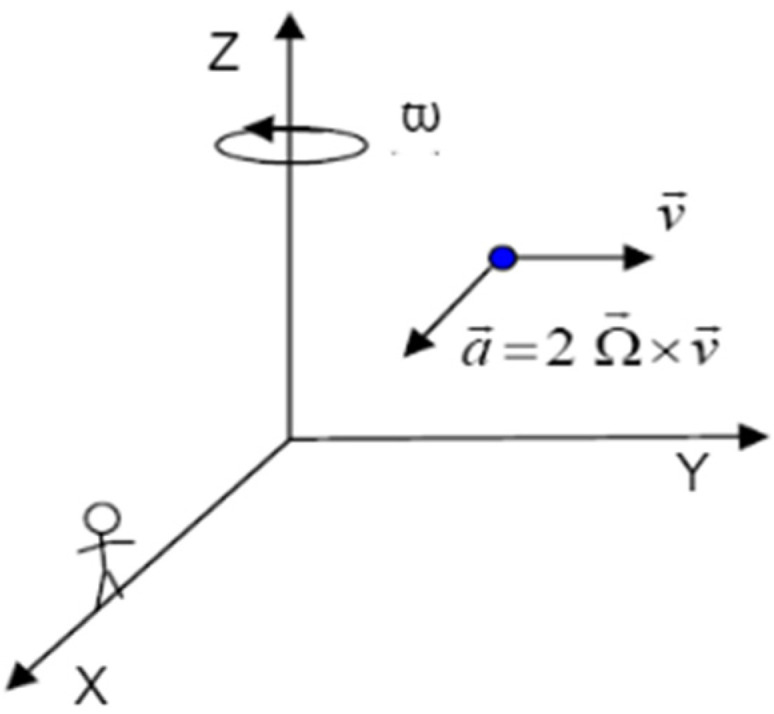
Perception of the Coriolis force.

To better understand this effect, consider the particle in Figure 2 [2], which moves at constant velocity $\vec{v}$ along the y-axis, with the observer on the x-axis looking at it. As reflected in the figure, the observer is located on the x-axis of the Cartesian coordinate system. In this coordinate system, if the rotation around the z-axis happens at angular velocity Ω, the observer notices a change in the direction of the particle relative to the x-axis with acceleration $\vec{v}\;\times \;2\vec{\Omega }$. The force resulting from this acceleration appears on a third axis, which is perpendicular to the axis of rotation and the velocity vector of the particle and whose value is proportional to the angular velocity along the z-axis. Although no real force is applied to the particle, from the observer’s point of view, the rotation of the reference frame creates a fictitious force that is directly proportional to the rotational speed. This is the operating principle of vibratory gyroscopes with different types of resonators. Finally, the Coriolis force is obtained using Equation (1), where m is the mass of the sensitive element, $\vec{v}$ is the velocity of the sensitive element and $\vec{\Omega }$ is the angular velocity. Since the Coriolis force is proportional to the velocity, increasing the excitation velocity of the sensitive element can lead to better sensitivity. However, as mentioned in the previous sections, the input velocity and changes in position of the HRG can be measured using the Coriolis force [23,24,25].

(1) $$\vec{F\;}=2m\vec{\Omega }\times \vec{v}$$

**Figure 3:**
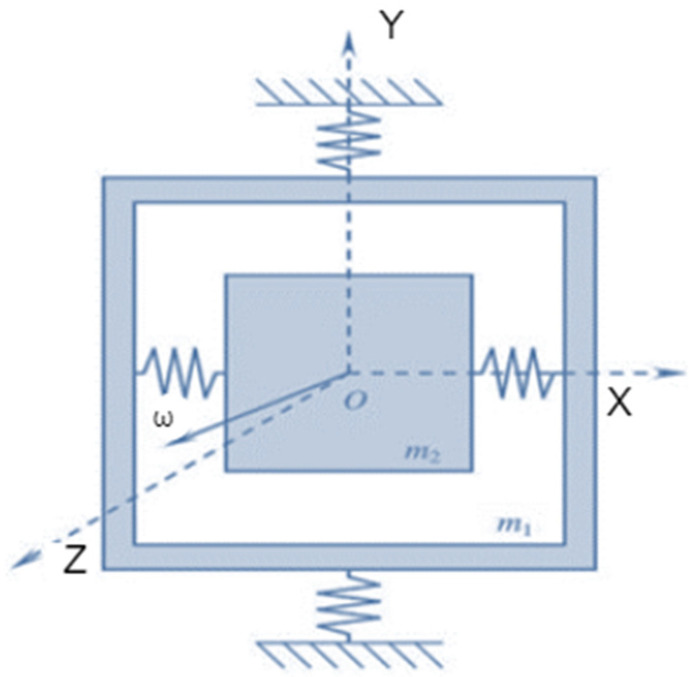
Linear movements of the proof mass.

The circular part of the resonator hemispherical shell constantly repeats “circular-horizontal elliptical” and “circular-vertical elliptical” motions during vibration. The point of maximum amplitude and the non-vibrating point are called “anti-nodal” and “nodal” points, respectively. HRG oscillation has two nodes and two anti-nodes during secondary resonance. Therefore, an HRG can be modeled with the damping system of spring oscillations and unbalanced mass exchange equations. Iso-elastic errors cause disentangled frequency and damping mismatch, resulting in an undesirable quality factor (Q). These are important problems that can lead to errors in HRG performance. Sensitive element orbiting refers to primary and secondary oscillations. In general, the orbiting sensitive element is referred to as gyroscope LL (primary and secondary linear motion). Figure 3 [2] shows a schematic of the operational structure of proof mass motions. In general, the sensitive element consists of a test mass $m_{2}$, a separate frame $m_{1}$ and a set of elastic elements (springs) to connect the masses to each other and to the base. In addition, the primary oscillations caused by excitation happen along the y-axis; therefore, the secondary oscillations occur along the x-axis. Hence, the z-axis is considered the sensitive axis.

### Three-Dimensional Finite Element Analysis

**Figure 4:**
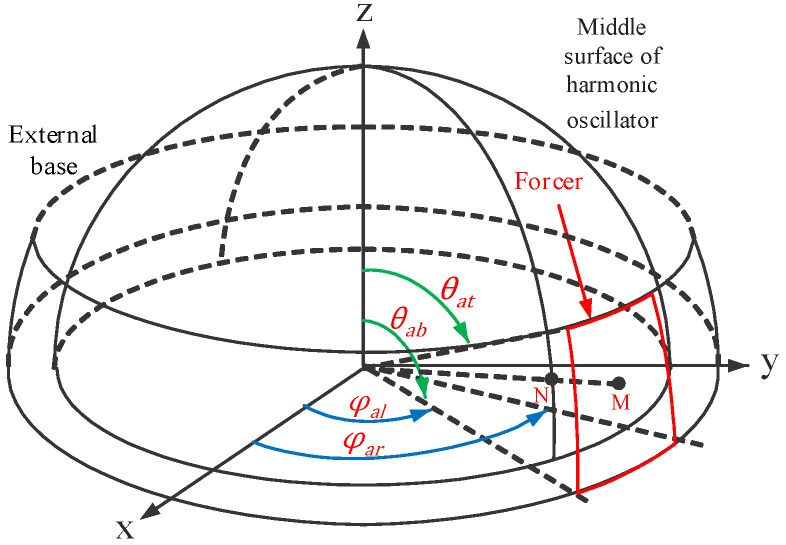
HRG drawn in spherical coordinates with capacitive excitation.

An HRG in a spherical coordinate system is shown in Figure 4. Constants E, ρ and μ are Young’s modulus, air density and Poisson’s ratio, respectively. R and h are the radius and thickness of the resonator, respectively. If the changes in the Coriolis force and the angular acceleration equations are combined, then the frequencies of the different HRG modes are given by [17,18]

(2) $$\omega =\sqrt[{\;}]{\;\frac{n^{2}\;{\left(n^{2}-1\right)}^{2}\;E\;\int_{\varphi 0}^{\varphi f}\;\frac{\tan^{2n}\left(\frac{\varphi }{2}\right)}{\sin^{3}\left(\varphi \right)\;}\;h^{3}d\varphi }{3r^{4}\left(1+\mu \right)\;\rho \int_{\varphi 0}^{\varphi f}\;\left(n^{2}+1+\sin^{2}\left(\varphi \right)+2n\cos\left(\varphi \right)\right)\times \sin\left(\varphi \right)\tan^{2n}\left(\frac{\varphi }{2}\right)h\;d\varphi }}$$

In Figure 4 [3], φ is the angle formed at a given point on the +x-axis, and θ is the angle formed at a given point on the +z-axis. In (2), ω is the frequency of the different excitation modes, and n is the number of excitation modes.

The resonator movements in a spherical three-dimensional coordinate system are given by [18,19,20]

(3) $$D\left(\begin{array}{c} \frac{\partial^{3}\omega }{\partial \theta^{3}}+\frac{\mu }{\sin{\left(\theta \right)}^{2}}\frac{\partial^{3}\omega }{\partial \theta \partial \varphi^{2}}+\frac{\cos\left(\theta \right)}{\sin\left(\theta \right)}\frac{\partial^{2}\omega }{\partial \theta^{2}}\\ -\frac{\cos\left(\theta \right)\left(1+\mu \right)}{\sin{\left(\theta \right)}^{2}}\frac{\partial^{2}\omega }{\partial \varphi^{2}}+\frac{\sin{\left(\theta \right)}^{2}-\cos{\left(\theta \right)}^{2}}{\sin{\left(\theta \right)}^{3}}\frac{\partial \omega }{\partial \theta } \end{array}\right)+D_{1}\left(\frac{1}{\sin{\left(\theta \right)}^{2}}\frac{\partial^{3}\omega }{\partial \theta \partial \varphi^{2}}-\frac{\cos\left(\theta \right)}{\sin{\left(\theta \right)}^{3}}\frac{\partial^{2}\omega }{\partial \varphi^{2}}\right)=R^{4}X$$

(4) $$D\left(\begin{array}{c} \frac{\partial^{3}\omega }{\partial \theta^{3}}\frac{1}{\sin{\left(\theta \right)}^{3}}+\frac{\mu }{\sin{\left(\theta \right)}^{\;}}\;\frac{\partial^{3}\omega }{\partial \theta^{2}\partial \varphi^{\;}}+\frac{\cos\left(\theta \right)}{\sin{\left(\theta \right)}^{2}}\;\frac{\partial^{2}\omega }{\partial \theta^{\;}\partial \varphi^{\;}}\\ +\frac{\cos\left(\theta \right)\left(1+\mu \right)}{\sin{\left(\theta \right)}^{2}}\frac{\partial^{\;}\omega }{\partial \varphi^{\;}} \end{array}\right)+D_{1}\left(\frac{1}{\sin{\left(\theta \right)}^{\;}}\;\frac{\partial^{3}\omega }{\partial \theta^{2}\partial \varphi^{\;}}+\frac{1^{\;}}{\sin{\left(\theta \right)}^{3}}\frac{\partial^{\;}\omega }{\partial \varphi^{\;}}\right)=R^{4}Y$$

(5) $$D\left(\begin{array}{c} \frac{\partial^{4}\omega }{\partial \theta^{4}}+\frac{1}{\sin{\left(\theta \right)}^{3}}\frac{\partial^{4}\omega }{\partial \varphi^{4}}+\frac{2\mu }{\sin{\left(\theta \right)}^{2}}\frac{\partial^{4}\omega }{\partial \theta^{2}\partial \varphi^{2}}\\ +2tg\left(\theta \right)\frac{\partial^{3}\omega }{\partial \theta^{3}}-\frac{2\mu \cos\left(\theta \right)}{\sin{\left(\theta \right)}^{3}}\frac{\partial^{3}\omega }{\partial \theta \partial \varphi^{2}}-\frac{1}{tg{\left(\theta \right)}^{2}}\frac{\partial^{2}\omega }{\partial \theta^{2}}\\ \frac{2\left(1+\mu \right)}{\sin{\left(\theta \right)}^{4}}\frac{\partial^{2}\omega }{\partial \varphi^{2}}+\left(\frac{2\cos\left(\theta \right)}{\sin\left(\theta \right)}+\frac{\cos\left(\theta \right)}{\sin{\left(\theta \right)}^{3}}\right)\frac{\partial \omega }{\partial \theta } \end{array}\right)+D_{1}(\frac{2}{\sin{\left(\theta \right)}^{2}}\frac{\partial^{4}\omega }{\partial \theta^{2}\partial \varphi^{2}}-\frac{2\cos\left(\theta \right)}{\sin{\left(\theta \right)}^{3}}\frac{\partial^{3}\omega }{\partial \theta \partial \varphi^{2}}\frac{2}{\sin{\left(\theta \right)}^{4}}\frac{\partial^{2}\omega }{\partial \varphi^{2}})$$

where

(6) $$D=\frac{2Eh^{3}}{3\left(1-\mu^{2}\right)}$$

(7) $$D_{1}=\frac{2Eh^{3}}{3\left(1+\mu^{2}\right)}$$

and R represents the radius of the resonator. After solving the equations above, the frequency of vibrations and displacements spotted by the sensor can be calculated and analyzed using (MATLAB v. 2014 b and COMSOL v. 5) software.

## 3. Designing

**Figure 5:**
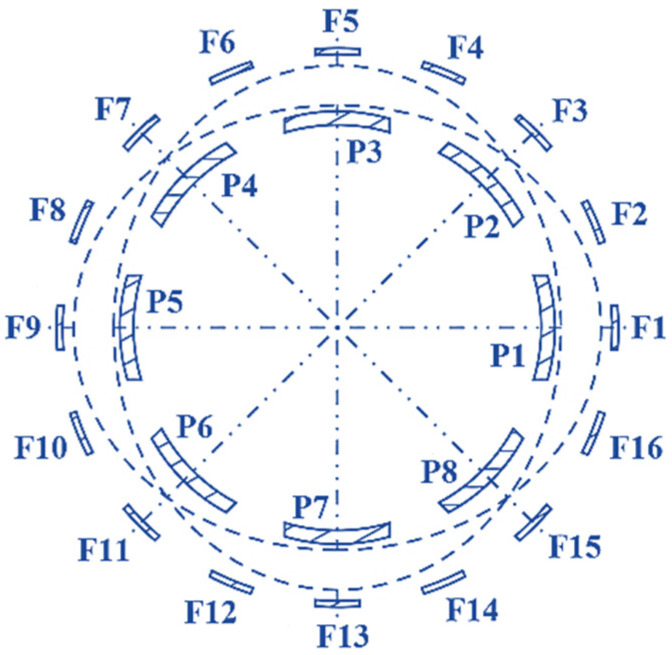
Placement of pickoff and forcer electrodes.

Figure 5 illustrates an HRG with capacitive drive. In this case, 16 forcer electrodes (those electrodes that apply the Lorentz force to the resonator) are located at equal distances of 360/16, or 22.5° (the distance of the electrodes is 20°, and the adjacent distance is 2.5°). In this model, eight pickoff electrodes (sensors) are used with distances equal to 360/8, or 45° (the distance of the electrode itself is 40°, and the adjacent distance is 5°). Table 2 shows the position of 16 forcer electrodes, while Table 3 shows the position of 8 pickoff electrodes. The voltage applied to the forcers is considered to be sinusoidal, with amplitude V_0_ and half of the frequency of the second-order resonance mode, which is given by

(8) $$V=\{\begin{array}{c} 0\;\;\;\;\;\;\;\;\;\;\;\;\;\;\;\;\;\;\;\;\;\;\;\;\;\;\;\;\;\;\;\;\;\;\;\;\;\;\;\;\;\;\;\;\;\;\;\;\;else\\ V_{0}\cos\left(\frac{\omega_{2}t}{2}\right)\;\;\;\;\varphi_{al}<\varphi <\varphi_{ar}\;\;;\;\;\theta_{at}<\theta <\theta_{ab} \end{array}\;$$

The frequency of the applied power is considered to be half of the resonant frequency of the resonator in the second mode, so second-order resonance does not occur. Since the spherical capacitor is in the micro-scale and this size of capacitor is very small compared with the radius of the resonator, it can be considered a flat capacitor. That is, the curvature of the resonator is ignored. Therefore, the electrostatic force applied to the resonator is given by

(9) $$F_{ES}=-\frac{\varepsilon_{0}V^{2}}{2d^{2}}=-\frac{\varepsilon_{0}V_{0}^{2}}{2d^{2}}\;u\left(\theta ,\varphi \right)\;\cos{\left(\frac{\omega_{2}t}{2}\right)}^{2}\;\;$$

where *u* is a binary variable to model the presence of an applied force to the related area and is given by

(10) $$u\left(\theta ,\varphi \right)=\{\begin{array}{c} \;0\;\;\;\;\;\;\;\;\;\;\;\;\;\;\;\;\;\;\;\;\;\;\;\;\;\;\;\;\;\;else\\ 1\;\;\;\;\;\;\;\varphi_{al}<\varphi <\varphi_{ar}\;\;;\;\;\theta_{at}<\theta <\theta_{ab} \end{array}$$

We then apply the following trigonometric conversion rule:

(11) $$\cos^{2}\left(x\right)=\frac{1}{2}\;\left(\cos\left(2x\right)+1\right)\;\;$$

The electrostatic force is simplified and is given by

(12) $$F_{ES}=-\frac{\varepsilon_{0}V^{2}}{2d^{2}}=-\frac{\varepsilon_{0}V_{0}^{2}}{4d^{2}}\;u\left(\theta ,\varphi \right)\;(\cos\left(\omega_{2}t\right)+1){\;}^{\;}\;$$

If the constant is not taken into account, the above is given by

(13) $$F_{ES}=-\frac{\varepsilon_{0}V^{2}}{2d^{2}}=-\frac{\varepsilon_{0}V_{0}^{2}}{4d^{2}}\;u\left(\theta ,\varphi \right)\;cos\left(\omega_{2}t\right)\;\;$$

Therefore, the useful force applied to the resonator only has a vertical component, and its tangential component is eliminated.

## 4. Calculating the Vibration Point following Vibration and Rotation

**Figure 6:**
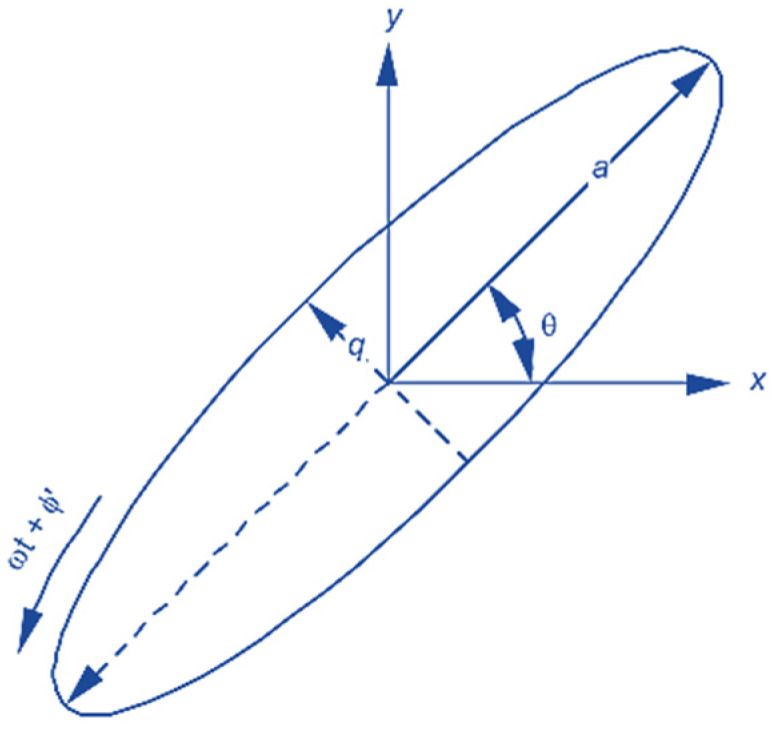
Rotating stimulated HRG gyroscope.

Considering the excitation caused by longitudinal and transverse oscillations in Coriolis vibratory gyroscopes, the gyroscope might be rotated at a certain speed for practical purposes. In this regard, all the coordinates of the given points on the HRG change position. One of the best ways to calculate the exact point of vibration is to use a transfer matrix. Suppose that the elliptical vertex (located on the larger diameter) has initial coordinates A(x, y). Now, if, according to Figure 6 [8], the ellipse or the excited gyroscope rotates at velocity ωt, point A changes position, which is given by

(14) $$\begin{array}{c} \ddot{x}+\omega^{2}x=0\\ \ddot{y}+\omega^{2}y=0 \end{array}$$

After solving (14), the following solutions are obtained:

(15) $$\begin{array}{c} x=a\cos\left(\theta \right)\cos\left(\omega t+\varphi \right)-q\;\sin\left(\theta \right)\sin\left(\omega t+\varphi \right)\\ x=a\sin\left(\theta \right)\sin\left(\omega t+\varphi \right)+q\;\cos\left(\theta \right)\cos\left(\omega t+\varphi \right) \end{array}$$

In (15), the impact of large- and small-diameter changes are ignored.

### 4.1. Analysis for Improving the Equations of Rotation and Vibration

**Figure 7:**
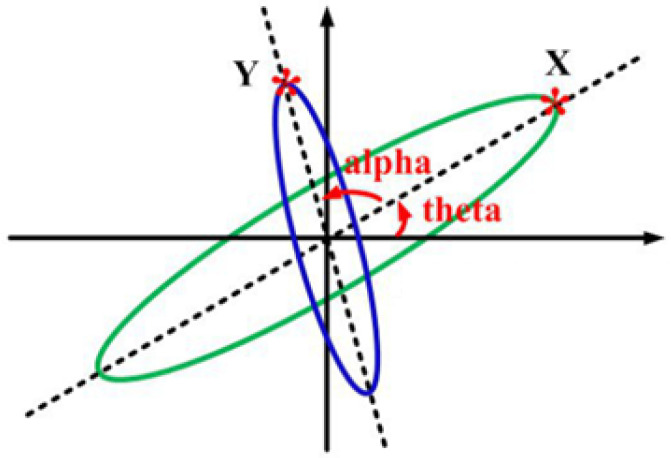
Gyroscope movement (of nodes and anti-nodes) in terms of rotation.

Suppose that the green ellipse in Figure 7 is a normal state of gyroscopic vibration. After a while, the gyroscope continues to rotate along with vibration, and point X changes to point Y. Therefore, the initial equation of the ellipse with a zero angle is given by

(16) $$\frac{x^{2}}{a^{2}}+\frac{y^{2}}{b^{2}}=1$$

The rotation and transformation matrix are applied as

(17) B=θA

where B(x’, y’) is the coordinate of converted point A(x, y) under rotation matrix θ. The rotation matrix is given by:

(18) $$\theta =\left[\begin{array}{cc} \cos\varphi & -\sin\varphi \\ \sin\varphi & \cos\varphi \end{array}\right]$$

where φ is the angle of rotation. Thus,

(19) $$A=\theta^{-1}B=\left[\begin{array}{cc} \cos\varphi & \sin\varphi \\ -\sin\varphi & \cos\varphi \end{array}\right]\left[\begin{array}{c} x^{\prime }\\ y^{\prime } \end{array}\right]=\left[\begin{array}{c} x^{\prime }\cos\varphi +y^{\prime }\sin\varphi \\ -x^{\prime }\sin\varphi +y^{\prime }\cos\varphi \end{array}\right]$$

Finally, the rotated ellipse can be formulated as

(20) $$x^{\prime 2}\left(\frac{\cos\varphi^{2}}{a^{2}}+\frac{\sin\varphi^{2}}{b^{2}}\right)+y^{\prime 2}\left(\frac{\sin\varphi^{2}}{a^{2}}+\frac{\cos\varphi^{2}}{b^{2}}\right)+x^{\prime }y^{\prime }\sin^{2}\varphi \left(\frac{1}{a^{2}}-\frac{1}{b^{2}}\right)=1$$

So, it could be verified as

(21) $$\begin{array}{c} if\;\varphi =0\;:\;\frac{x\prime^{2}}{a^{2}}+\frac{y\prime^{2}}{b^{2}}=1\\ if\;\varphi =90{}^{\circ}\;:\;\frac{y\prime^{2}}{a^{2}}+\frac{x\prime^{2}}{b^{2}}=1 \end{array}$$

which represent a horizontal ellipse and a vertical ellipse, respectively. Parameters *a* and *b* can be considered as functions of changes in the trigonometric ratios of the rotation angle and are given by

(22) $$\begin{array}{c} a=a\left(\varphi \right)=\frac{d_{max}+d_{min}}{2}+\left(\frac{d_{max}-d_{min}}{2}\right)\cos\left(4\varphi \right)[1-\cos^{2}\left(2\varphi \right)]\;\\ b=b\left(\varphi \right)=\frac{d_{max}+d_{min}}{2}-\left(\frac{d_{max}-d_{min}}{2}\right)\cos\left(4\varphi \right)[1-\cos^{2}\left(2\varphi \right)] \end{array}$$

### 4.2. Validation

In this section, the validation of the illustrated obtained ellipses (simultaneous vibration and rotation of HRG) in different positions using MATLAB software (2014b) is presented. Parameters $d_{max}=3.5\;\mathrm{cm}$ and $d_{min}=2.5\;\mathrm{cm}$ were considered for a gyroscope. So, in a circular state, it had a radius of 3 cm.

#### 4.2.1. φ=0°

**Figure 8:**
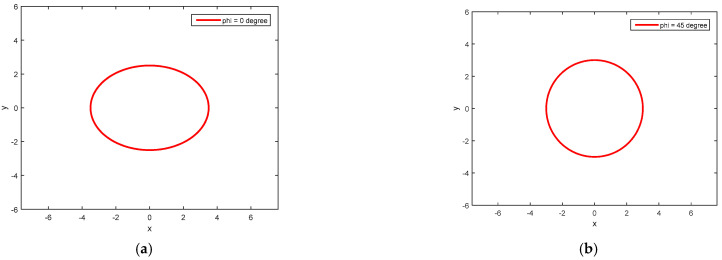

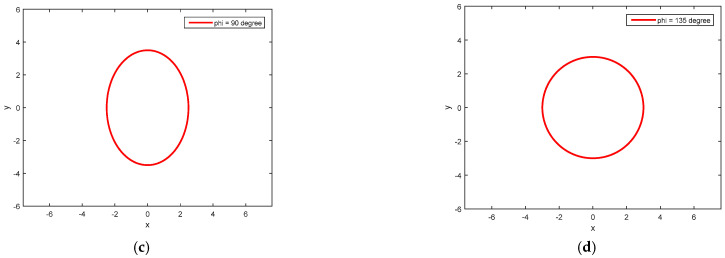
(**a**) Gyroscope display at φ = 0°; (**b**) gyroscope display at φ = 45°; (**c**) gyroscope display at φ = 90°; (**d**) gyroscope display at φ = 135°.

In the case of φ=0°, the ellipse is in the horizontal position. The diagram of this figure is shown in Figure 8a.

#### 4.2.2. φ=45°

At φ=45°, the ellipse is circular (nodes and abdomen overlap). The diagram is shown in Figure 8b.

#### 4.2.3. φ=90°

At φ=90°, the ellipse is in the vertical position, and Figure 8c represents it.

#### 4.2.4. φ=135°

At φ=135°, the ellipse is in a circular position (nodes and abdomen overlap), and it is represented in Figure 8d.

**Figure 9:**
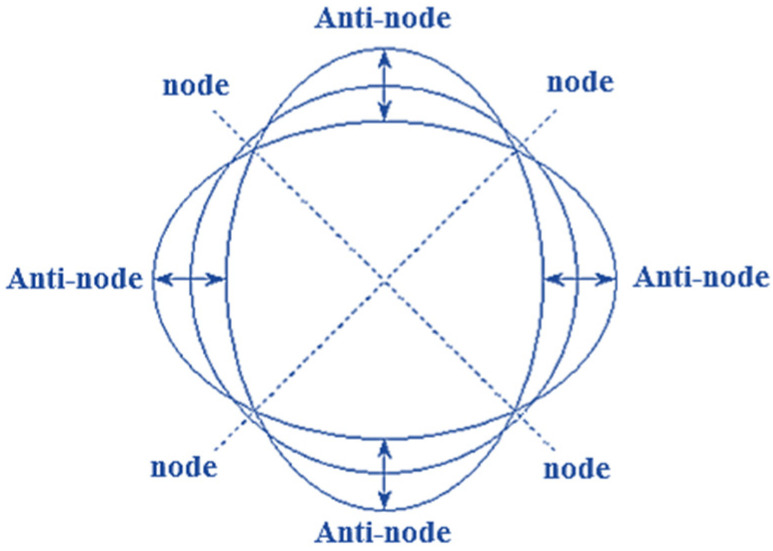
Gyroscope changing in nodes and anti-nodes in the second mode.

Figure 9 also shows a gyroscope in mode 2. By comparing Figure 8 and Figure 9, it can be concluded that the obtained relationships are quite correct.

## 5. Material Used in Electrodes

The material used in electrodes, their driving force and dimensions, along with the resonator characteristics, are shown in Table 4.

## 6. Drive Circuit

**Figure 10:**
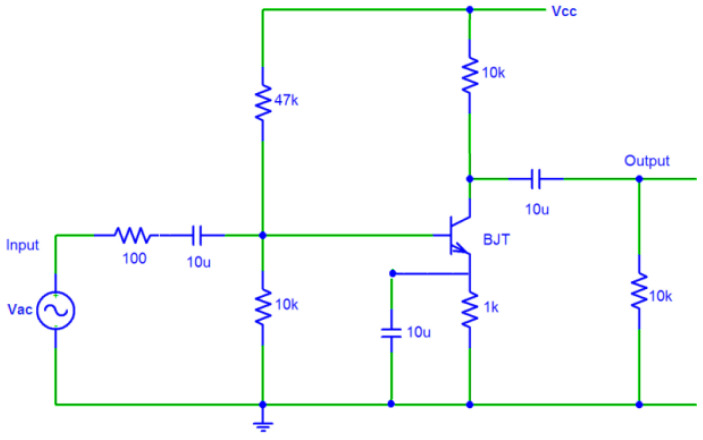
HRG gyroscope excitation circuit.

In COMSOL, the voltage cannot be directly applied to the forcing electrodes for exciting AC, which is a time-dependent signal, and an external circuit must be designed so that the electrode can be considered a part of that circuit. Therefore, the below excitation circuit can be used to apply voltage to the electrodes. This excitation circuit is depicted in Figure 10. V_cc_ was chosen as 10 V in order to turn on the transistor using the pulse needed by the gate. The AC voltage frequency was considered to be 3451.5 Hz to make sure that it was not equal to the second-order mode (to avoid resonance, set the applied power frequency to half the frequency of the second-order mode). The frequency of the second mode was also previously calculated as 6903 Hz with COMSOL.

**Figure 11:**
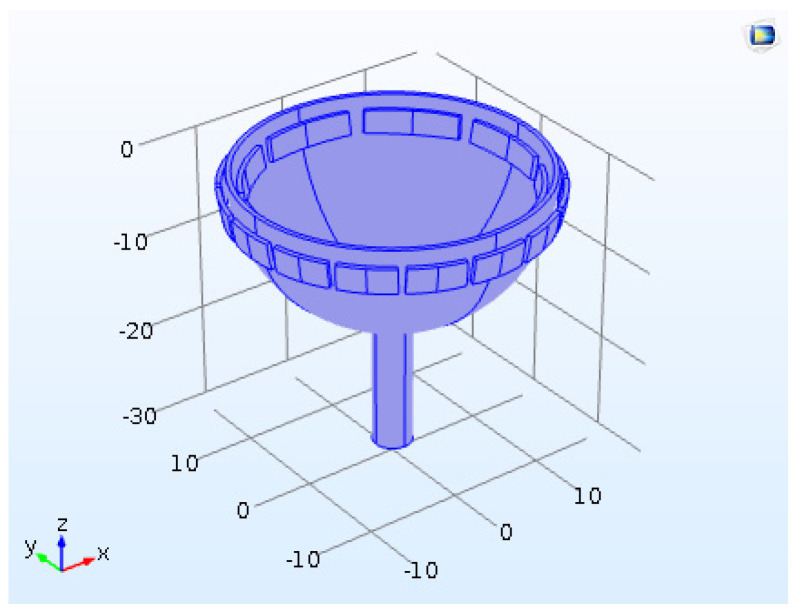
HRG simulated in COMSOL software.

**Figure 12:**
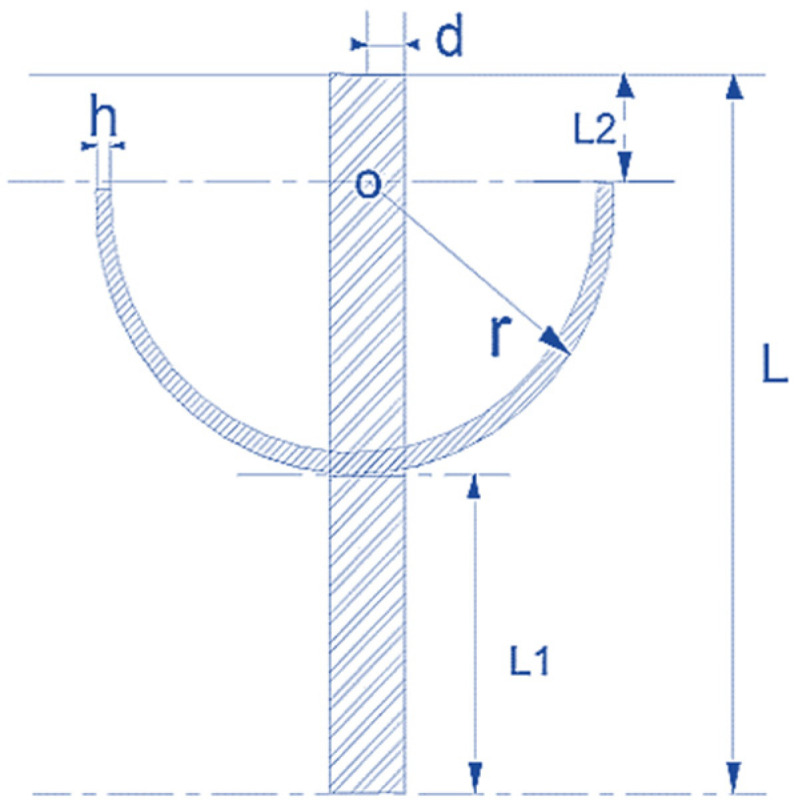
Two-dimensional view of HRG under simulation.

A three-dimensional illustration of an HRG device designed in COMSOL software is shown in Figure 11, where r=12.5 mm, h=0.8 mm, d=2.5 mm, L=39 mm and ρ=2651. A two-dimensional illustration of the HRG is shown in Figure 12.

## 7. Displacement and Excitation Frequency in Different Modes

**Figure 13:**
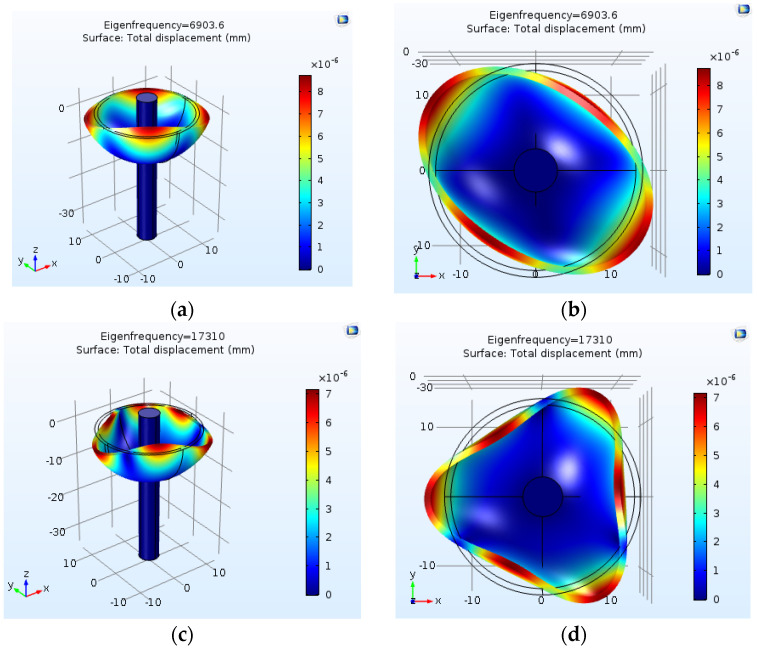

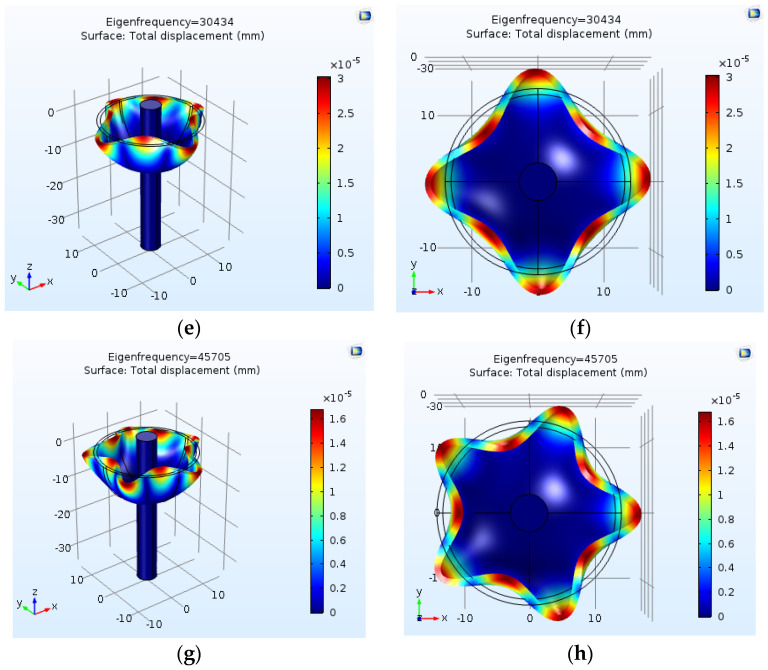
Display of placement and excitation frequency in the fourth and fifth modes. (**a**) Second mode—perspective; (**b**) second mode—view from the top; (**c**) third mode—perspective; (**d**) third mode—view from the top; (**e**) fourth mode—perspective; (**f**) fourth mode—view from the top; (**g**) fifth mode—perspective; (**h**) fifth mode—view from the top.

The excitation frequency and displacement of the HRG resonator in different modes were examined, and the results are presented in Table 5. The results in this table present the validation of the excitation frequency of HRG performed using COMSOL and (2). The 3D illustrations of the HRG in two–five operational modes are also shown in Figure 13.

## 8. Sensitivity Analysis of HRG Pickoffs and Forcers

Since the electrodes are distributed in a spherical space, increases in their length and width mean changes in azimuthal angle (φ) and polar angle (θ), which were calculated and are presented in Table 6 and Table 7. Table 6 examines the changes in (θ) angle, and Table 7 examines the changes in (φ) angle. As can be seen, the changes were only intended for electrode 1 or the reference electrode, and for 16 forcer electrodes and 8 pickoff electrodes. A 4-degree change was applied to the angles (2 degrees more and 2 degrees less than the base state), and the output results are announced as follows: The closer the forcer and pickoff electrodes were to the edge and the larger the surface they occupied, the higher the output voltage was. It should also be noted that the changes in the height of the pickoff and forcer electrodes were applied together.

The wider the electrodes were, the higher the voltage was, which was due to the increase in capacitance. Increasing the electrode width by two degrees led to a 4% increase in voltage.

## 9. Sensitivity Analysis of HRG Pickoffs and Forcers

Since the electrodes are distributed in a spherical space, increases in their length and width mean changes in azimuthal angle $\left(\varphi \right)$ and polar angle $\left(\theta \right)$, which were calculated and are presented in the following tables: Table 5 examines the changes in $\left(\theta \right)$ angle, and Table 6 examines the changes in $\left(\varphi \right)$ angle. As can be seen, the changes were only intended for electrode 1 or the reference electrode, and for 16 forcer electrodes and 8 pickoff electrodes. A 4-degree change was applied to the angles (2 degrees more and 2 degrees less than the base state), and the output results are announced as follows: The closer the forcer and pickoff electrodes were to the edge and the larger the surface they occupied, the higher the output voltage was. It should also be noted that the changes in the height of the pickoff and forcer electrodes were applied together.

## 10. Changes in Electrode Material in HRG

If the pickoff and forcer materials were changed, the voltages sensed varied. Table 8 was obtained using three widely used materials.

## 11. Changes in Electrode Material in HRG

When using piezo technology, the applied materials should have a high quality factor. Thus, achieving the ideal form increases costs, and piezo technology can also be achieved with low-cost materials. However, in this case, more control loops must be added to the circuit. One of the disadvantages of piezo technology is the heating of the resonator at the connection points of the piezo electrodes. This rise in heat can lead to deformation and lower efficiency in the long run. In addition, when piezo technology is used, the electrodes can also cause a change in shape, which may prevent the accurate calculation of the dynamics of the system. Therefore, some error may be accepted in this case. Piezo technology can be applied in both direct and alternating currents. However, due to the contact of the electrodes and the resonator, the low quality factor of the materials used to reduce costs, increased control loops, increased heat at the connection points, etc., this type of application comes with great disadvantages. The DC voltage range varies between 1 and 20 V, and the AC voltage range is not very limited and should be determined according to the generated heat. In order to solve these problems, we can either change the alignment of the electrodes (for example, skew or fragment them, etc.) or use AGC auxiliary signals to automatically control the gain of the system. However, this technology can also be applied in tuning-fork gyroscopes.

If the capacitive technology used is such that pickoff and forcer electrodes are placed next to each other and outside the resonator, direct voltage must be connected to the resonator in order for it to get biased. In addition, both direct and alternating voltages must be used. Direct voltage is needed for biasing electrodes, and alternating voltage is needed to apply the drive signal. In this case, the sense signal must occur within 45 degrees of the drive signal. Other available electrodes are also used to tune and balance the drive and sense electrical signals. The voltage range used here is sufficient for a DC voltage of about 0 to 100 volts and for an AC voltage of about 20 mV to 80 mV. Since the frequency of resonance is usually high, the heating impact (thermo-elastic power loss) is not considered. This problem should be addressed in design. The amount of QTED, or thermo-elastic heating, directly affects the choice of material. For example, silicon oxide is stronger than other materials. Polysilicon with a thickness of 1 micrometer can also have high capacity. Therefore, these two substances are good options in HRG manufacturing. Therefore, in this micromachined HRG technology, resonators and electrodes are made of silicon and nitrogen derivatives in order for them to have good performance in a semiconductor space.

Thus, if pickoffs and forcers are adjacent to each other, they can both be made of polysilicon. In fact, here, pickoffs and forcers were attached to the resonator and were made of the same material. The gap between adjacent electrodes was made of silicon nitride.

In combined piezocapacitive technology, reference signal tracking has been adequately described. The voltage range is about 5 V DC, which is transmitted to the piezo. Here, the capacitive electrodes are only used for capacitance sensing and act as pickoffs. Signal control and tuning are performed using eight external piezoelectric electrodes. This technology, despite its disadvantages in the first mode, may offer good results. The resonator was made of Ni43CrTi (3J53) (is an elastic alloy, which belongs to the precision alloy category), and piezoelectric elements PZT-5A were used. This material has high elasticity and quality factor, which requires a high number of control loops, as discussed above. The simultaneous use of piezo and capacitive technologies requires a lot of laboratory equipment compared with other methods, which is one of its disadvantages. In this case, energy signals are used to control the system.

In capacitive technology, the best HRG performance is only achieved if we use capacitive electrodes in which the pickoffs are placed inside the resonator and the forcers are placed outside of it, and using 16 electrodes allows us to easily apply drive and control signals to it at a certain amplitude and frequency. Piezoelectric disadvantages are also eliminated by the lack of connection between the electrodes and the resonator body. The only important obstacle in this case is the impact of the resonator on the system acceleration, which can be solved using the offered control methods. The power frequency is half the frequency of the second-order resonance; this completely eliminates resonance interference. Using different voltages at the same frequency and different amplitudes can also provide a suitable closed-loop control system, but these loops should not affect each other and should be decoupled to improve HRG performance. The decoupling of control loops is possible using modal analysis and determining the optimal factors in the Galerkin integral differential equations.

Finally, the best HRG performance is achieved when the selected optimal factors separate the control loops. Applying low-amplitude and low-frequency voltage is also one of the advantages of this system. In this system, accuracy is increased; errors caused by acceleration and noise are eliminated; and the cost and weight of the system are reduced. Using materials with a very high and ideal quality factor is no more required. Table 9 presents a summary of the above points.

## 12. Conclusions

In this paper, we present a general formulation for the analysis of two-dimensional and three-dimensional excitation of Coriolis vibratory gyroscopes based on capacitive excitation. The output simulation indicates a validation of the proposed equations, and this means accurate HRG modeling. Finite element simulations of the model using COMSOL software show that changes in parameters affecting the HRG resonance frequency can severely affect the optimal design. Sensitivity analyses of the physical and geometric characteristics of the gyroscope also indicate changes in the resonance frequency and output voltage sensed by the pickoffs. Pickoffs and forcers are also made of hard iron to prevent corrosion due to changes in electric and magnetic fields applied in the gyroscope. Sensitivity analyses of the planes of pickoff and forcer electrodes allow the user to choose the optimal design according to the measurable error rate. The results of the study also show that the use of the capacitive excitation technology provides better results than the traditionally used piezo technology and eliminates most of the disadvantages of piezo technology.

## Figures and Tables

**Table 1:** Comparison of VC, piezoresistive and piezoelectric technologies.

	Variable Capacitance (VC)	Piezoresistive	Piezoelectric
Functional frequency	0 Hz–6 kHz	0 Hz–6 kHz	3 Hz–30 kHz
acceleration	1 g–200 g	Up to 2000 g	Up to 20,000 g
Functional temperature	−55–175 °C	−55–125 °C	−200–700 °C
SNR	High	Fair	High
Measurement error	0.1%	2.1%	3.2%
Temperature sensitivity	No	Yes	Yes
Self-test	True internal self-test	No self-test	No self-test

**Table 2:** Position of pickoffs on HRG.

Electrode	φ		θ	
	From Angle (°)	To Angle (°)	From Angle (°)	To Angle (°)
Electrode 1	0	40	100	110
Electrode 2	45	85	100	110
Electrode 3	90	130	100	110
Electrode 4	135	175	100	110
Electrode 5	180	220	100	110
Electrode 6	225	265	100	110
Electrode 7	270	310	100	110
Electrode 8	315	355	100	110

**Table 3:** Position of pickoffs on HRG.

Electrode	φ		θ	
	From Angle (°)	To Angle (°)	From Angle (°)	To Angle (°)
Electrode 1	0	20	100	110
Electrode 2	22.5	42.5	100	110
Electrode 3	45	65	100	110
Electrode 4	67.5	87.5	100	110
Electrode 5	90	110	100	110
Electrode 6	112.5	132.5	100	110
Electrode 7	135	155	100	110
Electrode 8	157.5	177.5	100	110
Electrode 9	180	200	100	110
Electrode 10	202.5	222.5	100	110
Electrode 11	225	245	100	110
Electrode 12	247.5	267.5	100	110
Electrode 13	270	290	100	110
Electrode 14	292.5	312.5	100	110
Electrode 15	315	335	100	110
Electrode 16	337.5	357.5	100	110

**Table 4:** Material of electrodes, type of driving force and their dimensions.

Parameter	Material	Excitation	Dimension and Number
Peakoffs	Iron	--	8 electrodes with a longitudinal angle (φ) of 45° (40° of the electrode itself and 5° of adjacent distance) and a height angle (θ) of 10° with a radius of 14 mm and a thickness of 0.5 mm
Forcers	Iron	$V_{0}\cos\left(\frac{\omega_{2}t}{2}\right)$ where ω_2_ is the same excitation frequency in the second mode	16 electrodes with a longitudinal angle (φ) of 22.5 degrees (20 degrees of the electrode itself and 2.5 degrees of adjacent distance) and a height angle (θ) of 10 degrees with a radius of 16 mm and a thickness of 0.5 mm
Resonator	Fused Quartz	--	A hemisphere with an inner radius of 15 mm and an outer radius of 15.5 mm

**Table 5:** Comparison of calculated and measured frequencies.

Mode Number	Measured Frequency	Calculated Frequency
Second mode	6903 Hz	6899 Hz
Third mode	17,310 Hz	17,301 Hz
Fourth mode	30,434 Hz	30,427 Hz
Fifth mode	37,095 Hz	37,281 Hz

**Table 6:** Voltage sensed according to altitude location of electrodes (angle changes (θ)).

Kind of Sensitivity	(φ)		(θ)		$V_{Sense}{\left(V\right)}_{}$
	From	To	From	To	
Base-mode forcer	0	20	100	110	5.196
Base-mode peakoff	0	40			
Mode 1	0	21	100	110	5.223
Mode 2	0	22	100	110	5.401
Mode 3	0	19	100	110	5.179
Mode 4	0	18	100	110	5.161

**Table 7:** Voltage sensed according to transverse location of electrodes (angle changes (φ)).

Kind of Sensitivity	(φ)		(θ)		$V_{Sense}{\left(V\right)}_{}$
	From	To	From	To	
Base-mode forcer	0	20	100	110	5.196
Base-mode peakoff	0	40			
Mode 1	0	21	100	110	5.223
Mode 2	0	22	100	110	5.401
Mode 3	0	19	100	110	5.179
Mode 4	0	18	100	110	5.161

**Table 8:** Voltage sensed according to changes in electrode material.

Electrode material	$V_{Sense}\;(V)$
Mode I Soft Iron	5.197
Mode II Copper	5.246
Mode III Silicon Carbide	4.958

**Table 9:** Comparison of important gyroscopic parameters by type of stimulation.

Parameter	Piezo	Uniaxial Capacitor	Capacitor and Piezo	Biaxial Capacitor
Price	Cheap	Expensive	Expensive	Medium
Control ability	Yes	Yes	Yes	Yes
DC voltage range	1 V–20 V	0–100	About 5 V	Equal or greater than 100 V
AC voltage range	Equal or greater than 100 V	20 mV–80 mV	Equal or greater than 100 V	Equal or greater than 100 V
Weakness	Use of high-quality materials; many control loops; heating of the resonator at the junction of the piezo and, finally, deformation of the resonator in the long run; mechanical imbalance; shredding or skewing pickups and forceps for better control	Use of a DC voltage for resonator bias; requirement of tune and balance electrodes; occurrence of thermo-elastic losses and limited choice of material;	Piezo electrodes are only forcers, and capacitive electrodes are only pickups; use of many control loops	Application of high voltage; impact of gyroscope performance on system acceleration

## Data Availability

Not applicable.

## References

[1] Apostolyuk V. (2016). Coriolis Vibratory Gyroscopes.

[2] Usubamatov R. (2018). Inertial forces acting on a gyroscope. J. Mech. Sci. Technol..

[3] Pai P., Chowdhury F.K., Mastrangelo C.H., Tabib-Azar M. (2012). MEMS-based hemispherical resonator gyroscopes. Proceedings of the 2012 IEEE SENSORS Proceedings.

[4] Ranji A.R., Damodaran V., Li K., Chen Z., Alirezaee S., Ahamed M.J. (2022). Recent Advances in MEMS-Based 3D Hemispherical Resonator Gyroscope (HRG)—A Sensor of Choice. Micromachines.

[5] Jose K., Suh W., Xavier P., Varadan V. (2002). Surface acoustic wave MEMS gyroscope. Wave Motion.

[6] Bergh R.A., Lefevre H.C., Shaw H.J. (1981). All-single-mode fiber-optic gyroscope. Opt. Lett..

[7] Prestage J.D., Chung S., Le T., Beach M., Maleki L., Tjoelker R.L. One-Liter Ion Clock: New Capability for Spaceflight Applications. Proceedings of the 35th Annual Precise Time and Time Interval Systems and Applications Meeting.

[8] Xie H., Fedder G. (2003). Fabrication, characterization, and analysis of a DRIE CMOS-MEMS gyroscope. IEEE Sens. J..

[9] Chouvion B., McWilliam S., Popov A. (2018). Effect of nonlinear electrostatic forces on the dynamic behaviour of a capacitive ring-based Coriolis Vibrating Gyroscope under severe shock. Mech. Syst. Signal Process..

[10] Wang M., Cao H., Shen C., Chai J. (2018). A Novel Self-Calibration Method and Experiment of MEMS Gyroscope Based on Virtual Coriolis Force. Micromachines.

[11] Guo K., Wu Y., Zhang Y., Sun J., Xiao D., Wu X. (2020). Damping Asymmetry Trimming Based on the Resistance Heat Dissipation for Coriolis Vibratory Gyroscope in Whole-Angle Mode. Micromachines.

[12] Salzenstein P., Kuna A., Sojdr L., Chauvin J. (2010). Significant step in ultra-high stability quartz crystal oscillators. Electron. Lett..

[13] Lavrik N.V., Datskos P.G. (2019). Optically read Coriolis vibratory gyroscope based on a silicon tuning fork. Microsystems Nanoeng..

[14] Guerinoni L., Falorni L.G., Gattere G. (2017). Modelling Cross Axis Sensitivity in MEMS Coriolis Vibratory Gyroscopes. Multidiscip. Digit. Publ. Inst. Proc..

[15] Raspopov V.Y., Alaluev R.V., Ladonkin A.V., Likhosherst V.V., Shepilov S.I. (2020). Tuning and Calibration of a Coriolis Vibratory Gyroscope with a Metal Resonator to Operate in Angular Rate Sensor Mode. Gyroscopy Navig..

[16] Basarab M., Lunin B. (2021). Solving the Coriolis Vibratory Gyroscope Motion Equations by Means of the Angular Rate B-Spline Approximation. Mathematics.

[17] Xiao P., Qiu Z., Pan Y., Li S., Qu T., Tan Z., Liu J., Yang K., Zhao W., Luo H. (2020). Influence of Electrostatic Forces on the Vibrational Characteristics of Resonators for Coriolis Vibratory Gyroscopes. Sensors.

[18] Wen H., Daruwalla A., Liu C.-S., Ayazi F. (2018). A High-Frequency Resonant Framed-Annulus Pitch or Roll Gyroscope for Robust High-Performance Single-Chip Inertial Measurement Units. J. Microelectromechanical Syst..

[19] Price R.H. (2020). Gyroscopes simply explained with Coriolis pseudotorques. Am. J. Phys..

[20] Norouzpour-Shirazi A., Ayazi F. (2017). A Dual-Mode Actuation and Sensing Scheme for In-Run Calibration of Bias and Scale Factor Errors in Axisymmetric Resonant Gyroscopes. IEEE Sens. J..

[21] Mittapally G.K.M., Dantala D., Chhabra I., Kishore P., Rao N.V.N., Das K.C. (2018). Analysis of metallic hemispherical shell vibration modes for coriolis vibratory gyroscope. IOP Conf. Ser. Mater. Sci. Eng..

[22] Shu L., Yu Y., Yong Z., Rao Z., Ke S., Fei L., Zhang S., Wang Y. (2019). Negative Coriolis effect in piezoelectric metamaterials. J. Alloy. Compd..

[23] Jie D. (2018). Simulating the performance of ring-based coriolis vibrating gyroscopic sensors. Microsyst. Technol..

[24] Xiao P., Qiu Z., Luo Y., Pan Y., Qu T., Yang K., Luo H., Qin S. (2020). Influence of Temperature Variation on the Vibrational Characteristics of Fused Silica Cylindrical Resonators for Coriolis Vibratory Gyroscopes. Sensors.

[25] Ding X., Jia J., Qin Z., Ruan Z., Zhao L., Li H. (2019). A Lumped Mass Model for Circular Micro-Resonators in Coriolis Vibratory Gyroscopes. Micromachines.

[26] Ardakani M.H.A. (2019). Development of 3D High-Q Fused Quartz Micro Structures for Precision Coriolis Vibratory Gyroscopes. Doctoral Dissertation.

[27] Senkal D., Shkel A.M. (2020). Whole-Angle MEMS Gyroscopes: Challenges and Opportunities.

[28] Hodjat-Shamami M., Ayazi F. (2020). Eigenmode operation of piezoelectric resonant gyroscopes. Microsystems Nanoeng..

[29] Parween R. (2020). Significance of the Asymmetry of the Haltere: A Microscale Vibratory Gyroscope. Appl. Bionics Biomech..

[30] Hu Z., Gallacher B. (2018). A mode-matched force-rebalance control for a MEMS vibratory gyroscope. Sens. Actuators A Phys..

